# Cardiometabolic diseases, frailty, and healthcare utilization and expenditure in community-dwelling Chinese older adults

**DOI:** 10.1038/s41598-021-87444-z

**Published:** 2021-04-08

**Authors:** Ke Gao, Bo-Lin Li, Lei Yang, Dan Zhou, Kang-Xi Ding, Ju Yan, Ya-Jie Gao, Xiao-Rui Huang, Xiao-Pu Zheng

**Affiliations:** 1 Department of Cardiology, The First Affiliated Hospital of Xi’an Jiaotong University, 277 West Yanta Road, Xi’an, 710061 Shaanxi People’s Republic of China; 2 Key Laboratory of Molecular Cardiology of Shaanxi Province, Xi’an, Shaanxi China; 3 Department of Gastroenterology, The First Affiliated Hospital of Xi’an Jiaotong University, Xi’an, Shaanxi China; 4 Department of Cardiology, The First Affiliated Hospital of Xiamen University, Xiamen, Fujian China; 5 Department of Cardiology, The First Affiliated Hospital of Xinjiang Medical University, Wulumuqi, Xinjiang China

**Keywords:** Epidemiology, Health care, Cardiovascular diseases, Comorbidities

## Abstract

This study investigated associations between cardiometabolic diseases, frailty, and healthcare utilization and expenditure among Chinese older adults. The participants were 5204 community-dwelling adults aged at least 60 years from the China Health and Retirement Longitudinal Study. Five cardiometabolic diseases were assessed including hypertension, dyslipidemia, diabetes, cardiac diseases and stroke. Frailty status was based on five criteria: slowness, weakness, exhaustion, inactivity, and shrinking. Participants were deemed frailty if they met at least three criteria. As the number of cardiometabolic diseases increased, so did the prevalence of frailty, and the proportion of healthcare utilization, including outpatient visit and inpatient visit. Moreover, the total healthcare expenditure and the odds of catastrophic health expenditure were increased with the number of cardiometabolic disorders. After adjusting for covariates, cardiometabolic diseases were positively associated with higher odds of frailty, incurring outpatient and inpatient visit. And individuals with 2 or more cardiometabolic diseases had a higher odds of catastrophic health expenditure than persons with non-cardiometabolic disease. Participants who were frailty were more likely to report higher odds of healthcare utilization. These findings suggest that both cardiometabolic diseases and frailty assessment may improve identification of older adults likely to require costly, extensive healthcare.

## Introduction

With the escalating burden of aging populations worldwide, the prevalence of multimorbidity is likely to increase rapidly^1,2^. Compelling evidence shows that multimorbidity is common among older adults, with a prevalence ranged from 6.4 to 76.5% among individuals aged ≥ 60 years in China^3,4^. Among these chronic diseases, cardiometabolic diseases, including hypertension, dyslipidemia, diabetes, cardiac diseases and stroke, are the main components of multimorbidity^3,5–7^. It has been demonstrated that cardiometabolic components are strongly associated with adverse outcomes, such as poor quality of life^8,9^, high mortality^1,10,11^, and increased functional limitation^12^. Although China has a rapidly growing elderly population, its healthcare systems were designed mainly for managing people with single acute illness, rather than multiple complex diseases. In this case, even though China has a higher burden of multimorbidity due to its rapidly aging population, there is a paucity of research on the association between cardiometabolic diseases and healthcare utilization and expenditure among Chinese older adults.

Frailty, a geriatric syndrome characterized by decreased reserve and reduced resistance to stressors, is strongly associated with an increased risk of adverse health outcomes. Frailty was measured using the previously validated scale described by Fried et al.^13^ and Bandeen-Roche et al.^14^. This scale assesses 5 individual dichotomous domains, and an individual was classified as being frailty if they met 3 or more criteria. Frailty has been found to be associated with multimorbidity, mortality, falls, poorer health status, inpatient visit, and care home admission^15–18^. However, little evidence exists on the association of cardiometabolic diseases with frailty among Chinese older adults. Higher burdens of chronic non-communicable diseases are associated with higher healthcare utilization and expenditure^19–21^. Population aging is conservatively estimated to be responsible for a total healthcare cost of 263 billion CNY to the healthcare systems of China in 2050^22^. However, there is considerable individual variability in healthcare expenditure and utilization in aged populations and current multimorbidity measures have only a modest predictive validity for total healthcare expenditure^23^. Several studies have shown that frailty was associated with higher subsequent total healthcare expenditure and utilization after accounting for multimorbidity^24,25^. Hence, timely identification of frailty is important to stratify the healthcare utilization and expenditure among older adults.

To date, evidence on the associations between cardiometabolic diseases, frailty, and healthcare utilization and expenditure among older community-dwelling adults is scarce in China. Moreover, little attention has been given to the effect of a higher number of cardiometabolic disease components on healthcare utilization and catastrophic health expenditure. In this study, we used data from the nationally representative China Health and Retirement Longitudinal Study (CHARLS). We aimed to assess associations between cardiometabolic diseases, frailty and healthcare utilization among older community-dwelling Chinese adults. In addition, we investigated the level of healthcare expenditure associated with cardiometabolic diseases and frailty.

## Results

### Baseline characteristics of participants

The baseline characteristics of the study participants are shown in Table 1. The mean age of the 5204 participants was 67.8 years, and 2225 (42.7%) had cardiometabolic diseases. Individuals with cardiometabolic diseases were more often female and more likely to live in urban areas, had higher household income and education level, and had higher body mass index and higher prevalence of chronic conditions than non-cardiometabolic disease group. Supplementary Table 1 presents the prevalence of cardiometabolic disease components overall and by frailty category. Approximately 30.6% of the study participants were hypertension at their interview, while 9.7% reported having been diagnosed as dyslipidemia, 6.7% reported diabetes, 15.1% reported cardiac diseases, and 2.6% reported stroke.

**Table 1:** Baseline characteristics of participants according to cardiometabolic diseases status.

Characteristics	Overall (n = 5204)	Non-cardiometabolic diseases (n = 2979)	Cardiometabolic diseases (n = 2225)	*P* value
Age, y	67.8 ± 6.5	67.7 ± 6.6	68.0 ± 6.4	0.081
Male, n (%)	2644 (50.8)	1613 (54.1)	1031 (46.3)	< 0.001
**Education, n (%)**				< 0.001
Elementary school and below	4268 (82.0)	2536 (85.2)	1732 (77.8)	
Secondary school	843 (16.2)	401 (13.5)	442 (19.9)	
College and above	93 (1.8)	42 (1.3)	51 (2.3)	
Married (vs. others), n (%)	4137 (79.5)	2382 (80.0)	1755 (78.9)	0.338
Urban (vs. rural), n (%)	1102 (21.2)	455 (15.3)	647 (29.1)	< 0.001
**Smoking status, n (%)**				< 0.001
Current	1627 (31.5)	1047 (35.3)	580 (26.3)	
Previous	602 (11.6)	300 (10.1)	302 (13.7)	
Never	2942 (56.9)	1622 (54.6)	1320 (60.0)	
**Socioeconomic status, n (%)**				< 0.001
Tertile 1 (the poorest)	1710 (33.3)	1071 (36.5)	639 (29.1)	
Tertile 2	1711 (33.3)	1010 (34.4)	701 (31.9)	
Tertile 3 (the richest)	1714 (33.4)	855 (29.1)	859 (39.1)	
BMI, kg/m^2^	22.5 (20.2, 25.2)	21.6 (19.7, 24.1)	23.8 (21.3, 26.6)	< 0.001
**Self-reported diseases, n (%)**				
Lung disease	711 (13.7)	367 (12.3)	344 (15.5)	0.001
Liver disease	209 (4.0)	95 (3.2)	114 (5.2)	< 0.001
Kidney disease	322 (6.2)	156 (5.3)	166 (7.5)	0.001
Stomach disease	1157 (22.3)	644 (21.7)	513 (23.1)	0.224
Arthritis or rheumatism	1924 (37.0)	1018 (34.2)	906 (40.8)	< 0.001
Asthma	277 (5.3)	134 (4.5)	143 (6.4)	0.002

Data are shown as means ± standard deviation, median (interquartile range), or numbers (percentages).

### Association of cardiometabolic diseases with frailty

**Figure 1:**
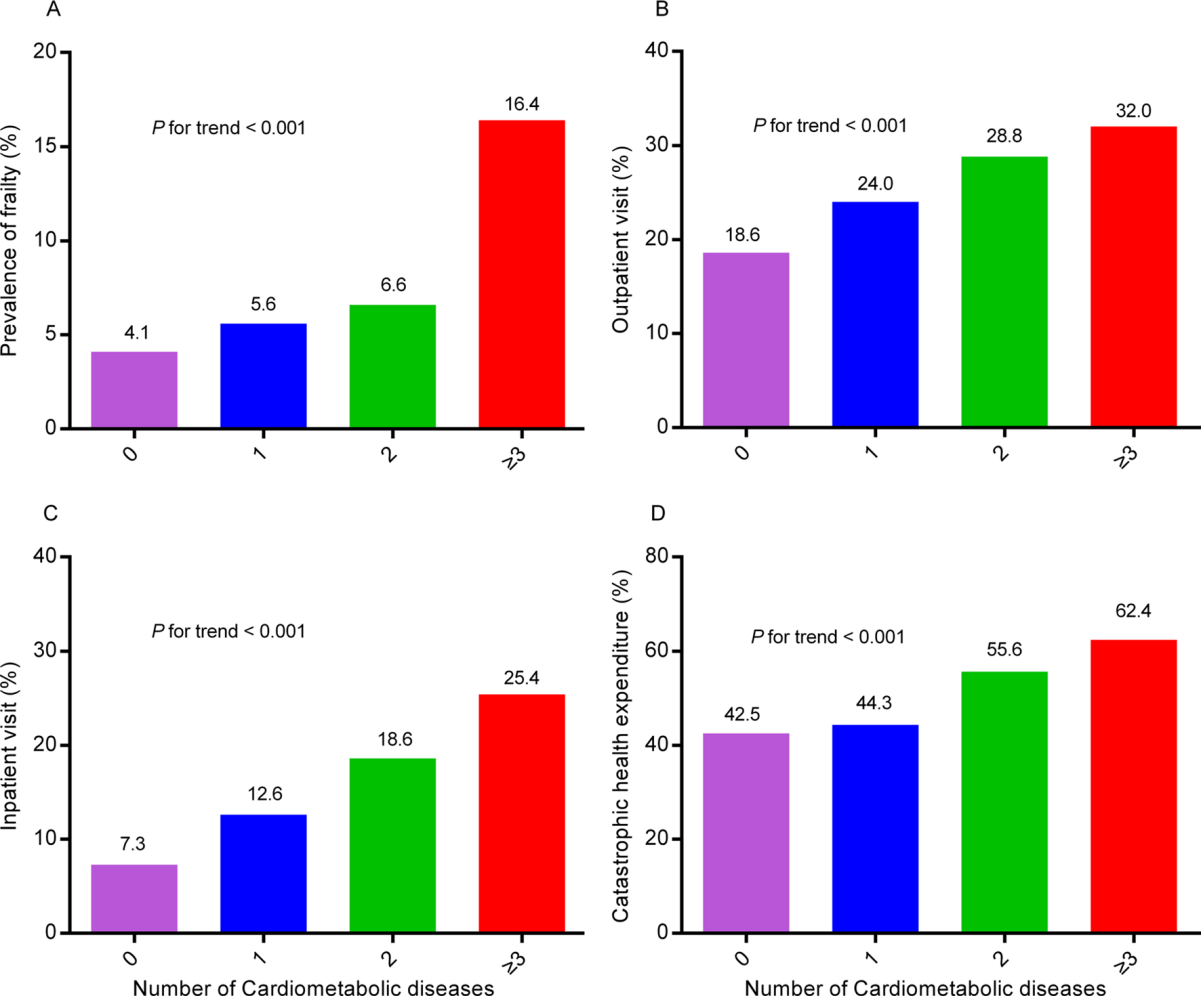
Prevalence of frailty, and healthcare utilization and expenditure by counts of cardiometabolic diseases. Column graph describing the prevalence of (**A**) frailty, (**B**) outpatient visit, (**C**) inpatient visit, and (**D**) catastrophic health expenditure, stratified by counts of cardiometabolic diseases.

The prevalence of frailty in total populations, non-cardiometabolic diseases group and cardiometabolic diseases group were 5.4%, 4.1%, 7.1%, respectively (Table 2). And frail persons had higher prevalence of all the components of cardiometabolic diseases than the non-frailty individuals (Supplementary Table 1). In addition, as the number of cardiometabolic diseases increased, so did the prevalence of frailty (*P* for trend < 0.001, Fig. 1A and Supplementary Table 2). Compared to non-cardiometabolic diseases, the presence of cardiometabolic diseases was significantly associated with higher odds of frailty (OR 1.482; 95% CI 1.234–1.779, *P* < 0.001, Table 3). We further found that as the number of cardiometabolic diseases increased, so did the odds of frailty.

**Table 2:** Frailty, healthcare utilization and expenditure by cardiometabolic diseases status.

Characteristics	Overall (n = 5204)	Non-cardiometabolic diseases (n = 2979)	Cardiometabolic diseases (n = 2225)	*P* value
Frailty, n (%)	279 (5.4)	122 (4.1)	157 (7.1)	< 0.001
**Healthcare utilization**				
Outpatient visit, n (%)	1136 (21.8)	554 (18.6)	582 (26.2)	< 0.001
Inpatient visit, n (%)	565 (10.9)	218 (7.3)	347 (15.6)	< 0.001
Number of outpatient visits				
Mean ± SD	0.5 ± 1.6	0.4 ± 1.4	0.6 ± 1.9	< 0.001
Median (IQR)	0 (0, 1)	0 (0, 1)	0 (0, 1)	< 0.001
Inpatient hospital days				
Utilization, n (%)	575 (11.0)	221 (7.4)	354 (15.9)	< 0.001
Mean ± SD	12 ± 10	11 ± 9	13 ± 10	0.087
Median (IQR)	10 (7, 15)	9 (6, 15)	10 (7, 15)	0.025
**Healthcare expenditure**				
Outpatient expenditure				
Utilization, n (%)	1085 (20.8)	534 (17.9)	551 (24.8)	< 0.001
Mean ± SD	120 ± 452	82 ± 235	157 ± 589	0.006
Median (IQR)	66 (15, 185)	46 (15, 157)	77 (19, 196)	< 0.001
Inpatient expenditure				
Utilization, n (%)	542 (10.4)	212 (7.1)	330 (14.8)	< 0.001
Mean ± SD	1223 ± 2004	1128 ± 2088	1284 ± 1948	0.377
Median (IQR)	615 (269, 1507)	615 (277, 1138)	615 (242, 1538)	0.069
Total healthcare expenditure				
Utilization, n (%)	1430 (27.5)	669 (22.5)	761 (34.2)	< 0.001
Mean ± SD	1561 ± 5269	1145 ± 2914	1927 ± 6667	0.005
Median (IQR)	1747 (790, 4230)	1661 (730, 4015)	1806 (808, 5653)	< 0.001
Catastrophic health expenditure				
Utilization, n (%)	1422 (27.3)	663 (22.3)	759 (34.1)	< 0.001
Yes, n (%)	664 (46.7)	282 (42.5)	382 (50.3)	0.003

Data are shown as means ± standard deviation, median (interquartile range), or numbers (percentages).

**Table 3:** Association of cardiometabolic diseases with frailty.

Cardiometabolic diseases	Model 1		Model 2	
	OR (95% CI)	*P* value	OR (95% CI)	*P* value
No (n = 2979)	Ref	Ref	Ref	Ref
Yes (n = 2225)	1.575 (1.316, 1.886)	< 0.001	1.482 (1.234, 1.779)	< 0.001
**Number of cardiometabolic diseases**				
0 (n = 2979)	Ref	Ref	Ref	Ref
1 (n = 1391)	1.331 (1.083, 1.636)	0.007	1.280 (1.039, 1.578)	0.020
2 (n = 590)	1.897 (1.449, 2.484)	< 0.001	1.698 (1.288, 2.238)	< 0.001
≥ 3 (n = 244)	2.979 (2.048, 4.333)	< 0.001	2.781 (1.903, 4.064)	< 0.001

Model 1 was adjusted for age, sex, residence, education level, marital status, smoking status, socioeconomic status and body mass index. Model 2 was adjusted as model 1 with further adjustment for lung disease, liver disease, kidney disease, stomach disease, arthritis or rheumatism, and asthma.

### Associations of cardiometabolic diseases, frailty status and healthcare utilization

All individuals had data on outpatient visit, inpatient visit and the number of outpatient visits. A total of 575 (11.0%) had data regarding inpatient hospital days over the past year. Persons with cardiometabolic diseases had higher prevalence of healthcare utilization, including outpatient visit and inpatient visit, and higher number of outpatient visits and length of stay than their counterparts (Table 2). Besides, the prevalence of outpatient visit, inpatient visit, and hospital stay were increased with higher number of cardiometabolic diseases (all *P* for trend < 0.001, Supplementary Table 2 and Fig. 1B,C).

**Figure 2:**
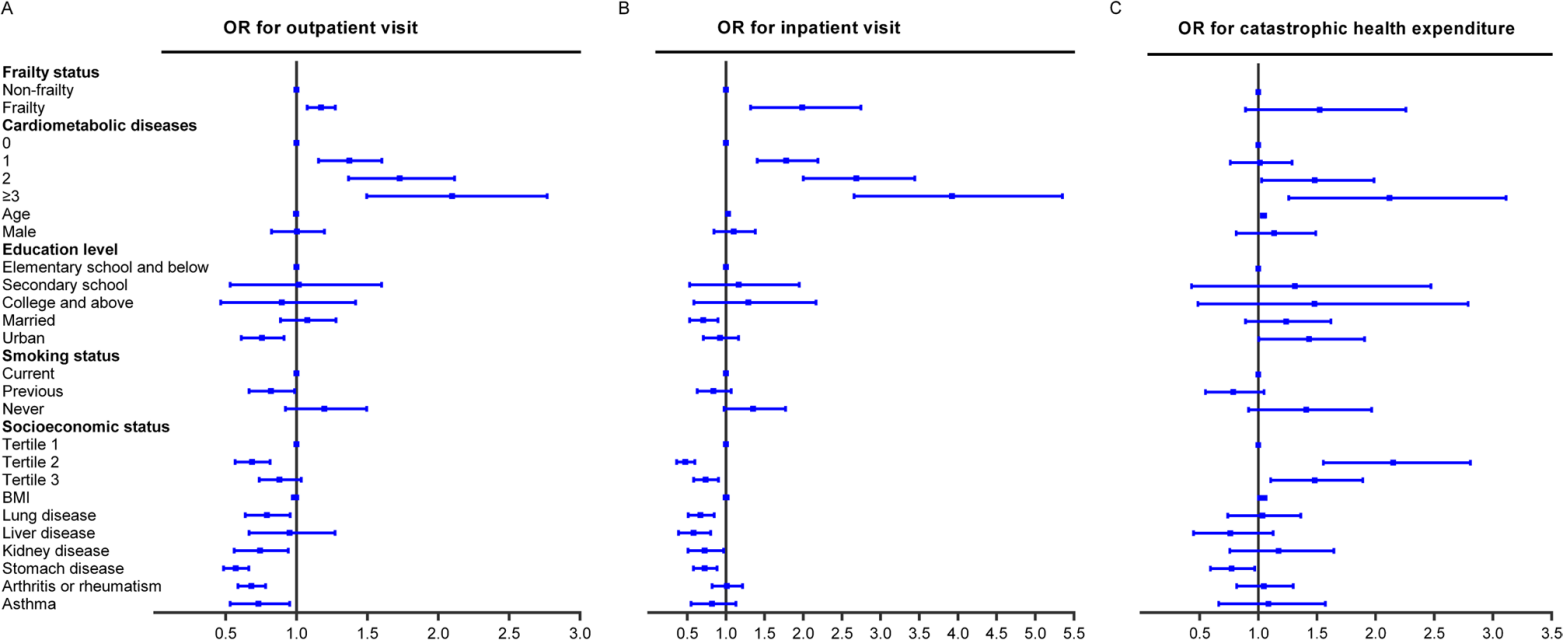
The association between cardiometabolic diseases, healthcare utilization and catastrophic health expenditure. Forest plots describing the associations of cardiometabolic diseases with (**A**) outpatient visit, (**B**) inpatient visit, and (**C**) catastrophic health expenditure.

After adjusting for all covariates, cardiometabolic diseases were positively associated with healthcare utilization, including incurring outpatient visit (OR 1.488; 95% CI 1.292–1.713, *P* < 0.001), and inpatient visit (OR 2.134; 95% CI 1.756–2.594, *P* < 0.001, Supplementary Table 3). Individuals with 1 cardiometabolic disease, 2 cardiometabolic diseases, and 3 or more cardiometabolic diseases had a 36.1% (95% CI 15.6%, 60.3%), 70.1% (95% CI 36.8%, 111.6%) and 103.5% (95% CI 49.5%, 176.9%) higher odds of outpatient visit than the non-cardiometabolic diseases, respectively (Fig. 2A). The same is true with inpatient visit: as the number of cardiometabolic diseases increased, so did the odds of inpatient visit (Fig. 2B). We also found that frailty was associated with higher odds of healthcare utilization, including outpatient visit (OR 1.171; 95% CI 1.075–1.275, *P* < 0.001), and inpatient visit (OR 1.902; 95% CI 1.318–2.744, *P* = 0.001, Fig. 2), after adjusted for multimorbidity and other potential confounders.

### The association between cardiometabolic diseases, frailty status and catastrophic health expenditure

A total of 1430 (27.5%) persons had data regarding total healthcare expenditure over the last year; 669 (22.5%) non-cardiometabolic diseases and 761 (34.2%) cardiometabolic disease persons had total healthcare expenditure, respectively (Table 2). On average, persons with cardiometabolic diseases had higher healthcare expenditure, including outpatient expenditure and total healthcare expenditure, than their counterparts (Table 2). Among individuals with cardiometabolic diseases, the median (IQR) of total healthcare expenditure increased from US $630 (308, 1646) for 1 cardiometabolic disease to US $1077 (461, 2307) for 2 cardiometabolic diseases to US $1538 (750, 2646) for persons with 3 or more cardiometabolic diseases (Supplementary Table 2). Besides, frail persons had higher median (IQR) of outpatient expenditure and total healthcare expenditure than non-frail individuals (Supplementary Table 4).

A total of 1422 participants (27.3%) had data regarding catastrophic health expenditure in the last year, including 663 (22.3%) non-cardiometabolic diseases and 759 (34.1%) cardiometabolic disease individuals. Persons with cardiometabolic diseases had a higher likelihood of catastrophic health expenditure than their counterparts (50.3% vs. 42.5%, *P* = 0.003, Table 2). And the prevalence of catastrophic health expenditure was increased with the number of cardiometabolic disorders (*P* for trend < 0.001, Supplementary Table 2 and Fig. 1D). After adjusting for all covariates, the presence of cardiometabolic diseases was not significantly associated with catastrophic health expenditure (OR 1.195; 95% CI 0.949–1.504, *P* = 0.130, Supplementary Table 5). However, individuals with 2 cardiometabolic diseases, and 3 or more cardiometabolic diseases had a 42.8% (95% CI 2.7%, 98.6%) and 98.1% (95% CI 26.0%, 211.3%) higher odds of incurring catastrophic health expenditure than persons with non-cardiometabolic diseases, respectively (Fig. 2C). In addition, frailty was not significantly associated with catastrophic health expenditure (OR 1.419; 95% CI 0.892–2.258, *P* = 0.140, Supplementary Table 5).

## Discussion

To our knowledge, using a large nationally representative survey of Chinese people, this study is the first attempt to examine the associations between cardiometabolic diseases, frailty, and healthcare utilization and expenditure**,** and the effect of different number of cardiometabolic diseases on the odds of frailty and catastrophic health expenditure among Chinese older adults. We found that cardiometabolic diseases were positively associated with higher odds of frailty and healthcare utilization. Importantly, the likelihood of frailty, healthcare utilization and catastrophic health expenditure were increased with higher number of cardiometabolic diseases. In addition, individuals with frailty were at higher odds of healthcare utilization after accounting for multimorbidity. Our findings provided the new evidence supporting the healthcare and economic effect of both cardiometabolic diseases and frailty, it also provides further justification for timely screening and identification of both cardiometabolic diseases and frailty as part of a comprehensive strategy to reduce the substantial healthcare and economic burden of non-communicable diseases.

In our study, cardiometabolic diseases are independently associated with higher odds of frailty, and as the number of cardiometabolic diseases increased, so did the odds of frailty. These findings are in line with previous investigations^7,15,26–28^, suggesting that some components of cardiometabolic diseases were associated with a higher risk of frailty. Abu et al.^7^ found that among older patients with atrial fibrillation, patients with a higher burden of all types of comorbid conditions were more likely to be frailty, and individuals with 5 or more cardiometabolic conditions had 58% higher odds of frailty than those with 2 or less cardiometabolic disorders. Hanlon et al.^15^ reported that frailty was strongly associated with multimorbidity in those with four or more long-term conditions among middle-aged and older adults, and patients with diabetes had 4 times higher odds of frailty that non-diabetes persons. A possible reason for this strong relationship between cardiometabolic diseases and frailty may be that with the presence of multiple cardiometabolic diseases, individuals who perceive their health status as being poor may have decreased resilience, less optimism regarding their health, and worsening general functional status and internal regulatory mechanism, all of which leading to an increased susceptibility to frailty and the onset of more chronic diseases^7,29^.

Several studies have suggested that physical multimorbidity was associated with higher odds of healthcare utilization in middle and high income countries^21,30,31^. Palladino et al.^30^ found that the number of chronic conditions was associated with greater healthcare utilization in both medical doctor visits and inhospitalization in European countries. Jankovic et al.^31^ observed that an increased number of non-communicable diseases was significantly associated with a higher utilization of health care services, including general practitioner, inhospitalization and length of stay, in Serbia. However, these studies have not considered the healthcare effects of frailty as described recently^24,25,32^, and some subjects were middle-aged adults. No studies have investigated the association between cardiometabolic diseases and healthcare utilization after adjusting for frailty. In the present study, we found that the odds of healthcare utilization, including outpatient visit and inpatient visit, were increased with the counts of cardiometabolic diseases after adjusting for frailty. Few previous studies have determined associations of the frailty phenotype with measures of healthcare burden in older populations^24,33^. We also found that frailty was positively associated with higher odds of healthcare utilization among Chinese older adults. These results indicate that both cardiometabolic diseases and frailty contribute to the increased healthcare utilization and may increase the burden of health systems. Assessment of frailty and cardiometabolic diseases in older people might facilitate identification of those at greatest risk of healthcare utilization, who would benefit most from intervention.

To date, no studies have assessed the associations between the cardiometabolic diseases, frailty, and catastrophic health expenditure among older adults. Zhao et al.^21^ reported that multimorbidity was associated with a significantly increased likelihood of catastrophic health expenditure among 50 years and older adults. In the present study, we found that the total healthcare expenditure was increased with the higher number of cardiometabolic diseases. However, the presence of cardiometabolic diseases was not significantly associated with catastrophic health expenditure. We further observed that individuals with 2 and 3 or more cardiometabolic diseases had a 42.8%, and 98.1% higher odds of catastrophic health expenditure than persons with non-cardiometabolic diseases, respectively. One possible explanation is that greater absolute numbers of cardiometabolic diseases are required to trigger the so-called catastrophic expenditure threshold for those with cardiometabolic diseases. This result suggests that people with higher number of cardiometabolic conditions might have more intensive healthcare use or use more expensive healthcare services than those with 1 or non-cardiometabolic disease. Recent studies have reported the association between the frailty and healthcare expenditure^22,24^. In our study, although frailty was not significantly associated with catastrophic health expenditure, we also found that frail persons had higher outpatient expenditure and total healthcare expenditure than non-frail individuals. This suggests that frailty cannot be a catastrophic disease, and not all variability in healthcare utilization and expenditure can be explained by multimorbidity. Our findings also provide new evidence on the growing financial and healthcare burden of cardiometabolic diseases in China. Considering the high healthcare burden and the various adverse outcomes associated with frailty and cardiometabolic conditions in older people, developing and implementing comprehensive interventions to identify or prevent both cardiometabolic diseases and frailty is crucial. Health systems need to shift from single-disease models to new methods of financing and service delivery to more effectively manage multimorbidity.

This study has several important strengths. We used a large and nationally representative sample, thereby allowing for broad generalizability of our findings to Chinese older adults regardless of disease counts and socioeconomic status. Moreover, to the best of our knowledge, this is the first study to explore the associations between cardiometabolic diseases, frailty, healthcare utilization and catastrophic health expenditure in China. Several limitations are worth mentioning: First, this study used observational data, which may have biased the observed associations by introducing confounding factors. To reduce such bias, models controlled for several factors; however, it was not possible to control for all potential confounding factors. Second, the amount of healthcare utilization and healthcare expenditures are self-reported, so subjective measures would lead to a mismatched estimate of the associations between cardiometabolic diseases, healthcare utilization and catastrophic health expenditure. Third, this study shares all the limitations of observational, single country studies, and our findings cannot be extrapolated to other countries with completely different culture and food habits. Finally, we were unable to establish a causal association of cardiometabolic diseases with frailty, healthcare utilization and expenditure because our study is cross-sectional.

## Conclusion

In conclusion, cardiometabolic diseases were independently associated with higher odds of frailty and healthcare utilization among older adults, and frailty was an independent determinant of increased healthcare utilization even after accounting for multimorbidity. Integration of both cardiometabolic diseases and frailty assessment in community-based physical examination and routine clinical practice may improve the identification of older adults requiring more costly and extensive healthcare. At the same time, prevention of cardiometabolic diseases should be taken as a factor in reducing the healthcare utilization and expenditures.

## Methods

### Data and participants

In this population-based, national-level study, we used baseline data (2011–2012) from the CHARLS (http://charls.pku.edu.cn/)^34^. Briefly, CHARLS collects high-quality data via one-to-one interviews with a structured questionnaire, from a nationally representative sample of Chinese residents aged 45 years or older, selected using multistage stratified probability-proportionate-to-size sampling. With a total response rate of 80.5% in the baseline survey, a total of 17,708 Chinese residents were enrolled. Further details about the recruitment strategy and study design of the CHARLS have been previously documented^34^. A total of 7681 participants were at least 60 years of older at baseline, 2477 patients were excluded because of missing data of cardiometabolic diseases (n = 56), cancer (n = 75), emotional or psychiatric problems (n = 123), memory-related disease (n = 192) and had data on 3 or less frailty components (n = 2031), resulting in a study population of 5204 participants who aged 60 years or older and had data at least 4 or more frailty components.

The Biomedical Ethics Review Committee of Peking University approved CHARLS (approval number: IRB00001052-11015), and all participants were required to provide written informed consent.

### Chronic conditions and cardiometabolic diseases

There were 11 chronic conditions included in this study. We ascertained 11 self-reported chronic conditions by asking “Have you been diagnosed with the following chronic conditions by a doctor” including hypertension, dyslipidemia, diabetes, chronic lung diseases, liver disease, cardiac diseases (including myocardial infarction, coronary heart disease, angina, heart failure, or other heart problems), stroke, kidney disease, stomach disease, arthritis or rheumatism, and asthma. We did not include individuals with self-reported cancer, psychiatric and memory related diseases due to potential recall bias. Participants with hypertension, dyslipidemia, diabetes, cardiac diseases, and history of stroke were identified based on self-reports of a physician’s diagnosis. The presence of one or more was considered as having cardiometabolic diseases^5,26^.

### Frailty

Frailty was measured by an adapted version of the physical frailty phenotype approach (PFP), which was previously constructed and validated in the CHARLS cohort^22,35^. The PFP included 5 criteria: shrinking, weakness, exhaustion, slowness and inactivity^13^. The shrinking criterion was met if the respondent self-reported loss of at least 5 kg in the previous year or currently had a body mass index of 18.5 kg/m^2^ or lower. The weakness criterion was met when handgrip strength, assessed as the maximum of 4 readings (2 for each hand) by a handheld dynamometer, was at or below the sex- and body mass index specific cutpoints. The exhaustion criterion was met if the participant answered “A moderate amount of time; 3 to 4 days” or “Most of the time; 5 to 7 days” when asked “How often during the last week did you feel this way?” to either of the 2 questions from the Center for Epidemiological Studies-Depression scale: “I could not get going” and “I felt everything I did was an effort.” The slowness criterion was met when gait speed, measured as the average of 2 timed walk tests over a 2.5-m course, was at or below the sex- and height-specific cut-points. Participants met criteria for inactivity if they self-reported that they did not walk 10 or more minutes continuously during a usual week. Individuals scoring ≥ 3 out of a total of 5 points were considered frailty. The construct validity and predictive validity of the PFP have been confirmed in several cohorts^36^.

### Healthcare utilization and expenditure

In CHARLS, individuals were asked about their healthcare utilization, including outpatient visit and inpatient visit services, via the following questions: “In the last month have you visited a public hospital, private hospital, public health center, clinic, or health worker’s or doctor’s practice, or been visited by a health worker or doctor for outpatient care ?”, “How many times did you visit these medical facilities in the past month?”, “Have you received inpatient care in the past year?” and “How many days did you stay in hospital over the past year?”^21^. CHARLS also collected information on how much respondents paid for their outpatient services during the past month and for inpatient visits over the past year. We multiplied the monthly spending by 12 to calculate the annual out of pocket spending for each person for outpatient services^21^. Healthcare expenditure was defined as the total amount of outpatient and inpatient expenses during the past year. In addition, we calculated healthcare expenditure in 2011 American currency using the exchange rate in 2011 (1 US $ = 6.5 CNY) for international comparisons^22^.

The same information was also collected for spouses of all participants. To calculate catastrophic health expenditures at the household level, we used the out of pocket spending data for spouses as well. We defined a household as incurring catastrophic health expenditures when out-of-pocket spending on health equalled or exceeded 40% of a household’s capacity to pay. Further details about the catastrophic health expenditure have been previously documented^21,37^. We defined a binary variable, which indicated whether the participant’s household had catastrophic health expenditures or not.

### Covariates

The study covariates included individual sociodemographic characteristics and lifestyle behaviours. Sociodemographic variables included age, sex, marital status (married and others), education (elementary school and below, secondary school, and college and above), residence (rural, urban), socioeconomic status tertiles. Lifestyle behaviours included body mass index, and smoking status (never, former and current smokers). We used annual per-capita household consumption spending as a proxy for socioeconomic status. We defined three socioeconomic groups on the basis of tertiles of per-capita household consumption expenditure (tertile 1, 0-3160 CNY; tertile 2, 3160-6329 CNY; tertile 3, 6329 CNY or more).

### Statistical analysis

Data are presented as means ± standard deviation or median and interquartile range for continuous variables and percentages for categorical variables. Continuous variables that had a normal distribution were evaluated using Student’s t-test or ANOVA, whereas the Mann–Whitney test or the Kruskal–Wallis test were used for non-normally distributed data. Categorical variables and frequencies were compared with the chi-squared test. Firstly, we compared the baseline characteristics, frailty, and healthcare utilization and expenditure according to cardiometabolic diseases status; and we also compared the prevalence of cardiometabolic disease components stratified by the frailty status. Second, logistic regression analysis was used to estimate the association between cardiometabolic diseases status and frailty, and the relationship of cardiometabolic diseases with healthcare utilization. Then, the association between cardiometabolic diseases and catastrophic health expenditure was also evaluated by binary logistic regression. We included age, sex, residence, education level, marital status, smoking status, socioeconomic status and body mass index in the Model 1. Model 2 was adjusted as model 1 with further adjustment for lung disease, liver disease, kidney disease, stomach disease, arthritis or rheumatism, asthma, cardiometabolic diseases and/or frailty status. Besides, to further explore the effects of different number of cardiometabolic diseases on frailty, healthcare utilization and catastrophic health expenditure, we created a variable with 4 categories: non-cardiometabolic disease, 1 disease, 2 diseases, and 3 or more cardiometabolic diseases. The relationships between cardiometabolic disease counts, frailty, healthcare utilization and catastrophic health expenditure were evaluated by logistic regression analysis. For logistic regression analysis, we report associations as odds ratios (OR) and 95% confidence interval (CI) after adjusted for all confounding factors. All statistical analysis was performed retrospectively with SPSS 25.0 (SPSS, Inc., Chicago, IL), and R version 3.5.1. In all cases, *P* < 0.05 was considered significant.

### Ethics statement

All participants provided informed consent, and the protocol was approved by the Ethical Review Committee of Peking University (approval number: IRB00001052-11015). All procedures performed in studies involving human participants were in accordance with the ethical standards of the institutional and/or national research committee and with the 1964 Helsinki declaration and its later amendments or comparable ethical standards.

## Supplementary Information

Supplementary Informations.

## Data Availability

The datasets generated for this study are available on request to the corresponding author.
