# Supplementary material for: Cardiometabolic diseases, frailty, and healthcare utilization and expenditure in community-dwelling Chinese older adults
Source: Sci Rep. 2021 Apr 8;11:7776. doi: 10.1038/s41598-021-87444-z (PMC8032763; doi:10.1038/s41598-021-87444-z)
Supplement: Supplementary file 1 — Supplementary Informations. [file 41598_2021_87444_MOESM1_ESM.docx]

**Cardiometabolic diseases, frailty, and** **healthcare utilization and expenditure in community-dwelling Chinese older adults**

Ke Gao, Bo-Lin Li, Lei Yang, Dan Zhou, Kang-Xi Ding, Ju Yan, Ya-Jie Gao, Xiao-Rui Huang, Xiao-Pu Zheng**^*^**

**Supplementary Table 1**. Prevalence of cardiometabolic disease components by category of frailty status

| **Characteristics** | **Overall**  **(n=5204)** | **Non-frailty**  **(n=4925)** | **Frailty**  **(n=279)** | ***P*-value** |
| --- | --- | --- | --- | --- |
| Hypertension | 1593 (30.6) | 1476 (30.0) | 117 (41.9) | *<*0.001 |
| Dyslipidemia | 504 (9.7) | 459 (9.3) | 45 (16.1) | *<*0.001 |
| Diabetes | 351 (6.7) | 317 (6.4) | 34 (12.2) | *<*0.001 |
| Cardiac disease | 784 (15.1) | 708 (14.4) | 76 (27.2) | *<*0.001 |
| Stroke | 133 (2.6) | 116 (2.4) | 17 (6.1) | *<*0.001 |

Data are shown as numbers (percentages).

**Supplementary Table 2**. Frailty, and healthcare utilization and expenditure by counts of cardiometabolic diseases

| **Characteristics** | **Number of Cardiometabolic diseases** | | | | ***P*-value** |
| --- | --- | --- | --- | --- | --- |
|  | **0**  **(n=2979)** | **1**  **(n=1391)** | **2**  **(n=590)** | **≥3**  **(n=244)** |  |
| Frailty | 122 (4.1) | 78 (5.6) | 39 (6.6) | 40 (16.4) | *<*0.001 |
| **Healthcare Utilization** |  |  |  |  |  |
| Outpatient visit, n (%) | 554 (18.6) | 334 (24.0) | 170 (28.8) | 78 (32.0) | *<*0.001 |
| Inpatient visit, n (%) | 218 (7.3) | 175 (12.6) | 110 (18.6) | 62 (25.4) | *<*0.001 |
| Number of outpatient visits |  |  |  |  |  |
| Mean ± SD | 0.4 ± 1.4 | 0.6 ± 1.7 | 0.8 ± 2.3 | 0.7 ± 1.6 | *<*0.001 |
| Median (IQR) | 0 (0, 1) | 0 (0, 1) | 0 (0, 1) | 0 (0, 1) | *<*0.001 |
| Inpatient hospital days |  |  |  |  |  |
| Utilization, n (%) | 221 (7.4) | 178 (12.8) | 110 (18.6) | 66 (27.0) | *<*0.001 |
| Mean ± SD | 11 ± 9 | 11 ± 10 | 13 ± 9 | 15 ± 10 | *<*0.001 |
| Median (IQR) | 9 (6, 14) | 8 (6, 15) | 10 (7, 15) | 14 (9, 16) | *<*0.001 |
| **Healthcare Expenditure** |  |  |  |  |  |
| Outpatient expenditure |  |  |  |  |  |
| Utilization, n (%) | 534 (17.9) | 316 (22.7) | 162 (27.5) | 73 (29.9) | *<*0.001 |
| Mean ± SD | 82 ± 235 | 86 ± 190 | 282 ± 1000 | 191 ± 412 | *<*0.001 |
| Median (IQR) | 19 (8, 54) | 23 (8, 77) | 46 (11, 92) | 62 (31, 154) | *<*0.001 |
| Inpatient expenditure |  |  |  |  |  |
| Utilization, n (%) | 212 (7.1) | 163 (11.7) | 105 (17.8) | 62 (25.4) | *<*0.001 |
| Mean ± SD | 1128 ± 2088 | 930 ± 1559 | 1607 ± 2322 | 1669 ± 2039 | 0.012 |
| Median (IQR) | 461 (215, 1076) | 462 (230, 1077) | 769 (323, 1615) | 1076 (461, 1585) | *<*0.001 |
| Total healthcare expenditure |  |  |  |  |  |
| Utilization, n (%) | 669 (22.5) | 420 (30.2) | 232 (39.3) | 109 (44.7) | *<*0.001 |
| Mean ± SD | 1145 ± 2914 | 1137 ± 2326 | 3095 ± 11016 | 2480 ± 5155 | *<*0.001 |
| Median (IQR) | 698(307, 1811) | 630 (308, 1646) | 1077 (461, 2307) | 1538 (750, 2646) | *<*0.001 |
| Catastrophic expenditure |  |  |  |  |  |
| Utilization, n (%) | 663 (22.3) | 418 (30.1) | 232 (39.3) | 109 (44.7) | *<*0.001 |
| Yes, n (%) | 282 (42.5) | 185 (44.3) | 129 (55.6) | 68 (62.4) | *<*0.001 |

Data are shown as means ± standard deviation, median (interquartile range), or numbers (percentages).

**Supplementary Table 3**. Association of cardiometabolic diseases, frailty status and healthcare utilization

|  | **Model 1** | | **Model 2** | |
| --- | --- | --- | --- | --- |
|  | **OR (95% CI)** | ***P*-value** | **OR (95% CI)** | ***P*-value** |
| **Outpatient Visit** | | | | |
| Cardiometabolic diseases |  |  |  |  |
| No (n = 2979) | Ref. | Ref. | Ref. | Ref. |
| Yes (n = 2225) | 1.574 (1.374, 1.804) | *<*0.001 | 1.488 (1.292, 1.713) | *<*0.001 |
| Number of Cardiometabolic diseases |  |  |  |  |
| 0 (n = 2979) | Ref. | Ref. | Ref. | Ref. |
| 1 (n = 1391) | 1.414 (1.206, 1.659) | *<*0.001 | 1.361 (1.156, 1.603) | *<*0.001 |
| 2 (n = 590) | 1.879 (1.521, 2322) | *<*0.001 | 1.701 (1.368, 2.116) | *<*0.001 |
| ≥3 (n = 244) | 2.218 (1.641, 2.998) | *<*0.001 | 2.035 (1.495, 2.769) | *<*0.001 |
| Frailty status |  |  |  |  |
| Non-frailty (n = 4925) | Ref. | Ref. | Ref. | Ref. |
| Frailty (n = 279) | 1.282 (1.181, 1.390) | *<*0.001 | 1.171 (1.075, 1.275) | *<*0.001 |
| **Inpatient Visit** | | | | |
| Cardiometabolic diseases |  |  |  |  |
| No (n = 2979) | Ref. | Ref. | Ref. | Ref. |
| Yes (n = 2225) | 2.218 (1.831, 2.686) | *<*0.001 | 2.134 (1.756, 2.594) | *<*0.001 |
| Number of Cardiometabolic diseases |  |  |  |  |
| 0 (n = 2979) | Ref. | Ref. | Ref. | Ref. |
| 1 (n = 1391) | 1.773 (1.424, 2.207) | *<*0.001 | 1.752 (1.404, 2.188) | *<*0.001 |
| 2 (n = 590) | 2.844 (2.181, 3.708) | *<*0.001 | 2.624 (2.000, 3.442) | *<*0.001 |
| ≥3 (n = 244) | 4.123 (2.921, 5.820) | *<*0.001 | 3.770 (2.656, 5.352) | *<*0.001 |
| Frailty status |  |  |  |  |
| Non-frailty (n = 4925) | Ref. | Ref. | Ref. | Ref. |
| Frailty (n = 279) | 2.041 (1.433, 2.908) | *<*0.001 | 1.902 (1.318, 2.744) | 0.001 |

Model 1 was adjusted for age, sex, residence, education level, marital status, smoking status, socioeconomic status and body mass index. Model 2 was adjusted as model 1 with further adjustment for lung disease, liver disease, kidney disease, stomach disease, arthritis, rheumatism, asthma and cardiometabolic diseases or frailty status.

**Supplementary Table 4**. Healthcare expenditure by category of frailty status

| **Characteristics** | **Non-frailty**  **(n=4925)** | **Frailty**  **(n=279)** | ***P*-value** |
| --- | --- | --- | --- |
| Outpatient expenditure |  |  |  |
| Utilization, n (%) | 1010 (20.5) | 75 (26.9) | 0.011 |
| Mean ± SD | 118 ± 459 | 140 ± 377 | 0.656 |
| Median (IQR) | 61 (15, 170) | 96 (37, 308) | *<*0.001 |
| Inpatient expenditure |  |  |  |
| Utilization, n (%) | 502 (10.2) | 40 (14.3) | 0.027 |
| Mean ± SD | 1201 ± 1997 | 1412 ± 2074 | 0.457 |
| Median (IQR) | 615 (307, 1430) | 484 (106, 2576) | 0.847 |
| Total healthcare expenditure |  |  |  |
| Utilization, n (%) | 1332 (27.0) | 98 (35.1) | 0.003 |
| Mean ± SD | 1529 ± 5293 | 1881 ± 5028 | 0.473 |
| Median (IQR) | 1676 (767, 4115) | 2333 (922, 5644) | *<*0.001 |
| Catastrophic expenditure |  |  |  |
| Utilization, n (%) | 1325 (26.9) | 97 (34.8) | 0.004 |
| Yes, n (%) | 611 (46.1) | 53 (54.6) | 0.104 |

Data are shown as means ± standard deviation, median (interquartile range), or numbers (percentages).

**Supplementary Table 5**. The association between cardiometabolic diseases, frailty and catastrophic health expenditure

|  | **Model 1** | | **Model 2** | |
| --- | --- | --- | --- | --- |
|  | **OR (95% CI)** | ***P*-value** | **OR (95% CI)** | ***P*-value** |
| Cardiometabolic diseases |  |  |  |  |
| No (n = 2979) | Ref. | Ref. | Ref. | Ref. |
| Yes (n = 2225) | 1.207 (0.961, 1.516) | 0.106 | 1.195 (0.949, 1.504) | 0.130 |
| Number of Cardiometabolic diseases |  |  |  |  |
| 0 (n = 2979) | Ref. | Ref. | Ref. | Ref. |
| 1 (n = 1391) | 0.989 (0.762, 1.284) | 0.933 | 0.990 (0.761, 1.289) | 0.941 |
| 2 (n = 590) | 1.481 (1.070, 2.050) | 0.018 | 1.428 (1.027, 1.986) | 0.034 |
| ≥3 (n = 244) | 1.983 (1.266, 3.106) | 0.003 | 1.981 (1.260, 3.113) | 0.003 |
| Frailty status |  |  |  |  |
| Non-frailty (n = 4925) | Ref. | Ref. | Ref. | Ref. |
| Frailty (n = 279) | 1.367 (0.868, 2.153) | 0.177 | 1.419 (0.892, 2.258) | 0.140 |

Model 1 was adjusted for age, sex, residence, education level, marital status, smoking status, socioeconomic status and body mass index. Model 2 was adjusted as model 1 with further adjustment for lung disease, liver disease, kidney disease, stomach disease, arthritis, rheumatism, asthma and cardiometabolic diseases or frailty status.
